# Freeform Perfusable Microfluidics Embedded in Hydrogel Matrices

**DOI:** 10.3390/ma11122529

**Published:** 2018-12-12

**Authors:** Gabriela Štumberger, Boštjan Vihar

**Affiliations:** IRNAS—Institute for development of advanced applied systems, Valvasorjeva 42, 2000 Maribor, Slovenia; gabi.stumberger@gmail.com

**Keywords:** freeform, hydrogel, gelatine, microfluidics, FRESH, bioprinting, vascularization

## Abstract

We report a modification of the freeform reversible embedding of suspended hydrogels (FRESH) 3D printing method for the fabrication of freeform perfusable microfluidics inside a hydrogel matrix. Xanthan gum is deposited into a CaCl_2_ infused gelatine slurry to form filaments, which are consequently rinsed to produce hollow channels. This provides a simple method for rapid prototyping of microfluidic devices based on biopolymers and potentially a new approach to the construction of vascular grafts for tissue engineering.

## 1. Introduction

Due to the required high proximity of cells to blood vessels, the fabrication of vascular structures has enormous impact on the engineering of tissues and organs. However, the required resolution, structural integrity and simultaneous biocompatibility is yet to be achieved, thus, the fabrication of thick vascular tissues is still the main challenge to solve in tissue engineering [1,2,3]. Since the adoption of additive manufacturing for bio-medical research purposes [4,5] 3D bioprinting has evolved rapidly and was also attempted for use in vascularization strategies. Several approaches to solve this have already been made, including subtractive or additive methods, micro-patterning with photolithography, induced angiogenesis, etc. [2,6,7]. While existing techniques show a lot of promise, many challenges remain to be resolved. These include transfer from 2D to 3D structures, mechanical integrity of tissues with a high density of hollow tubes and shear stresses due to fluid flow, or resolution [2,6]. Kolesky et al. (2016) have successfully managed to create thick tissues with 3D vasculature; however, the process is limited in its geometry to an even mesh in a cuboid structure [8]. In this work, we propose a new approach on how to create freeform, perfusable channels embedded in a gelatine matrix, based on freeform reversible embedding of suspended hydrogels (FRESH) by Hinton et al., 2015 [9]. The FRESH method allows precise deposition of material inside a gelatine matrix (slurry), which offers support for the printed material and consequently allows the fabrication of almost any shape or form. The matrix is prepared by washing and blending gelatine granules, to produce a finely particulate slurry, such that the nozzle can move between the particles without disturbing the already deposited material. As the base is infused with Ca^2+^ ions, ion-polymerizing substances such as alginate are encapsulated and cross-linked directly upon deposition. After printing, the matrix is liquefied by heating and the printed structure can be liberated from the matrix [9]. The aim of this work was to adapt the above mentioned process to 3D print sacrificial material into the hydrogel matrix, which in contrast to the initial FRESH method is kept for further experimentation, leaving empty spaces in the printed pathway. This will allow the fabrication of complex microfluidic devices from biocompatible hydrogels, which promises new applications in vascular tissue engineering (e.g., by fabricating scaffolds with large internal channel volumes), as well as bio-compatible lab-on-a-chip devices. Complex microfluidic devices made from biocompatible materials will be made possible through very precise channel fabrication with channel diameters equivalent to the nozzles, possibly below 100 μm with the appropriate needles (G30 or above).

## 2. Materials and Methods

### 2.1. Preparing the Materials

The to be printed ‘vessel filament’ was prepared by dissolving 0.1 g of xanthan-gum (Herbana d.o.o.) in 20 mL of distilled water and stirring over night at room temperature. 75 μL of Royal-Blue 4001 ink (Pelikan AG, Berlin, Germany) were added to the liquid, to improve the visibility of the later printed filament. For the matrix, gelatine granules from porcine skin (Sigma-Aldrich, Saint Louis, MO, USA) were ground using a coffee grinder for 10 min and sifted through a mesh filter. Filters with 90, 140, 200 and 250 µm pore sizes were tested in the process. The obtained powder was used for further preparation of the matrix. 2 g of the gelatine powder were soaked in a 50 mM CaCl_2_ solution at room temperature and stirred for 1 min. The stirring was stopped and the soaked granules were let to set at the bottom of the container, then the supernatant was removed. Fresh CaCl_2_ solution was added and the process was repeated 2 more times, until the supernatant was completely clear. Air bubbles were removed from the solution in a desiccator under vacuum and the final supernatant was removed. The slurry was heated to 32 °C and mixed in a 4:1 ratio with a 10% gelatine solution (in 100 mM CaCl_2_ at 32 °C) and transferred to the printing container. Excess liquid was removed by capillary imbibition using a paper towel.

### 2.2. Printing

G-codes were prepared manually from vectorised images of the target geometries. Printing was performed on the Vitaprint (irnas.eu/vitaprint) using PlanetCNC software (Planet CNC TNG 2018, PlanetCNC d.o.o., Ljubljana, Slovenia) with a feedrate of 500 mm/min. As the nozzle, blunt-end G27 needles were used (inner diameter 0.21 mm).

### 2.3. Curing and Rinsing

**Figure 1:**
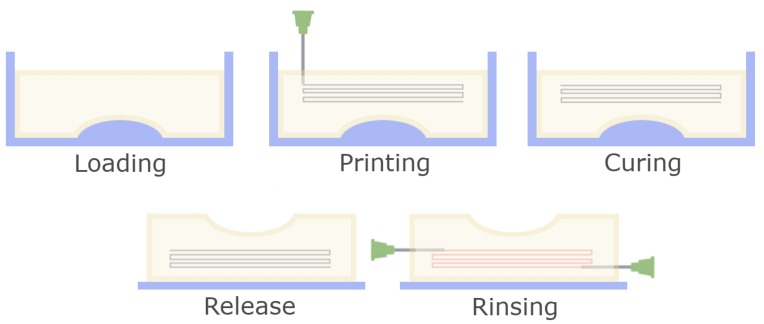
Schematic overview of the fabrication process for 3D structures. After the slurry is loaded into the mould, a channel pathway is printed using a fine nozzle. Then, the matrix is cured to create a homogenous, stable structure. Afterwards the device is released from the mould and the printed sacrificial material is removed by rinsing.

After printing the filament and matrix were let to rest at room temperature for 30 min to ensure graduate and even cross linking of the structure and then transferred to 6 °C overnight, for the matrix to harden completely. After this stage the structure could optionally be released from the mould and transferred to a handling surface for perfusion tests. The printed filament was removed by rinsing in the following manner: Blunt-end G21 needles were inserted into the matrix so the respective tip was positioned adjacently to the beginning or end of the 3D printing pathway. Water was manually injected through one needle using a syringe, pushing the printed material along the pathway towards the second needle and out of the solid matrix. A schematic of the protocol is shown in Figure 1.

### 2.4. Reproducing Channel Fabrication in an Alginate Matrix

For the method to be useful in a broad range of applications it should be compatible with a wide spectrum of materials. To determine if this was achievable, microfluidic fabrication was additionally attempted in an alginate-based matrix. First, alginate granulate needed to be produced, which was achieved by the protocol described by Poncelet in 1992 [10]. 20 mL of 1% Alginate solution in deionized H_2_O were mixed with 1mL of 0.5 M Ca-Citrate suspension and emulsified in 100 mL sunflower oil. During constant stirring on a magnetic stirrer the oil was acidified with 80 μL of glacial acetic acid to cross-link the alginate/Ca-citrate droplets. After stirring for 5 min, the emulsion was transferred to 150 ml of 50 mM CaCl_2_, 1% Tween solution. Granulate was extracted and rinsed with additional CaCl_2_/Tween solution until all residual oil droplets were removed before further use. Granulate was resuspended in 100 mL of dH_2_O for 30 min, drained and the washing process was repeated twice, and finally again with 10 mM NaCl. The suspension was drained and mixed with a 2% alginate solution in 10 mM NaCl in a 4:1 ratio. Two PMMA frames with inner dimensions of 20 × 40 × 4 mm were screwed together with a wet filter paper (Whatman 1) placed in between to function as the bottom of the printing container. The slurry was transferred to the upper frame and excess liquid was removed by capillary imbibition using a paper towel. Channel printing was performed as described above using xanthan gum. The PMMA container was placed into a pool to swim on 50 mM NaCl solution overnight at 6 °C to gently dissolve the alginate granulate. Afterwards the 50 mM NaCl solution was carefully exchanged with 50 mM CaCl_2_ solution and cured for 24 h at 6 °C.

## 3. Results and Discussion

### 3.1. Slurry Parameters

**Figure 2:**
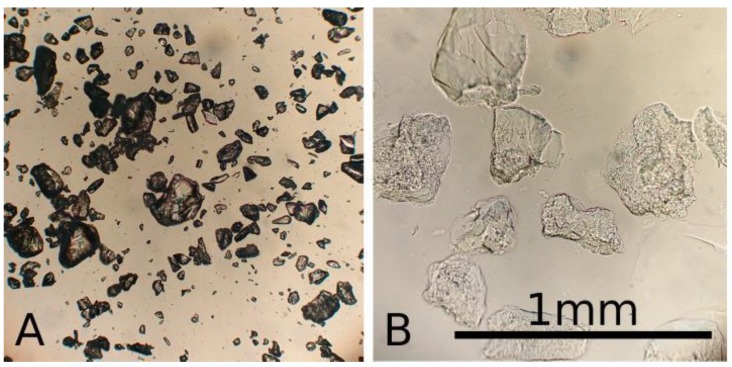
Microscopic image of gelatine granules when dry (**A**) and soaked (**B**) in 100 mM CaCl_2_ solution, compared to a reference bar of 1 mm.

Due to grinding, the matrix particles were irregularly shaped, also, soaking increased particle size significantly, see Figure 2. Matrix particle size had a strong impact on structural stability of the matrix, as well as the channel shape fidelity and perfusion. Finer matrix particles resulted in better vessel resolution and a more consistent shape. On the other hand, larger particles showed greater structural stability of the finished prototypes. 

The goal was to create a matrix with sufficient structural stability to support 3D structures while maintaining a perfusable vessel network. Particles <140 microns were suitable for 2D printing of perfusable vessels; however, for 3D structures they lacked sufficient structural stability. Best results for 3D channel printing were achieved with particles in size range of 90–200 µm mixed 1 + 3 with a 10% gelatine solution. In this constellation the fabricated vessels also showed good perfusion, see below.

### 3.2. Geometry

Fabrication of hydrogel microfluidics was tested on three levels of fabrication intricacy: simple two-dimensional devices, simple three-dimensional devices and complex three-dimensional devices, all of which should fulfill the following requirements:

- Repeatability
- Even perfusion through all channels
- Compatibility with various materials

**Figure 3:**
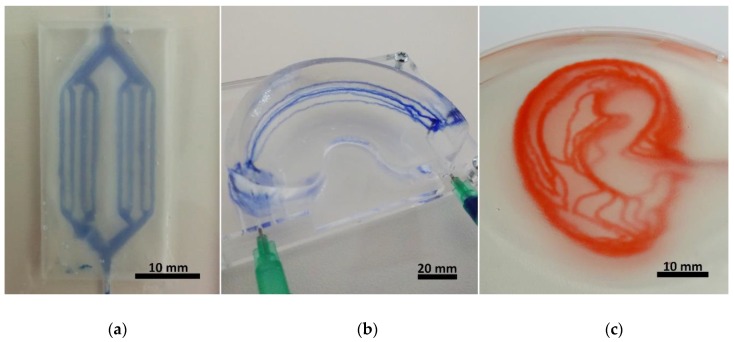
Photographs of the fabricated hydrogel microfluidic devices in increasing levels of complexity, made in a gelatine matrix. (**a**) shows a 2D branched geometry directly after fabrication, made in a 20 × 40 × 4 mm frame, with the channels printed at the height of 2 mm. (**b**) exhibits 5 arched channels which meet at the two ends of a meniscus shaped structure. The whole gelatinous structure (60 × 35 × 15 mm) was stable enough to keep its shape while still allowing perfusion (the channels were perfused with blue ink after releasing from mould). In (**c**) 3D earlobe shaped channels are shown, perfused with red ink. The channels are joined at the inlet/outlet points in the middle-right side of the structure, and then diverge into 9 layers, 1 mm apart. The projected earlobe is 58 mm long and 36 mm wide.

To create 2D branched channels, samples were fabricated inside a 4 mm thick acrylic plate with a 20 × 40 mm well, which was filled with the gelatine slurry and lateral inlet/outlet channels with 1mm diameter for perfusion. A simple branched geometry was designed, making a single stroke at the thinnest branches, a double when two are added together, and so on. Before printing the microfluidics frame was placed on the printer and the starting position (x,y,z) = (0,0,0) was set for the nozzle tip to begin at the inlet position in the well. For the simple 3D geometry a silicone mould was fabricated in the shape of a knee meniscus negative with inner dimensions of 55 × 35 × 12 mm. The mould was filled with the slurry and 6 relatively shifted parallel arcs were written to emulate a vessel system of the “vascular meniscus zone”. After curing the structure was removed from the mould to test the stability of the gelatine structure in addition to the perfusion. The structure was transferred to a perfusion stage with two installed G21 needles piercing the meniscus model and connecting to the arc starting points on each side. Finally, a simplified vessel system of the human earlobe was modelled to test channels with complex geometries in 3D. The slurry was transferred into a PP petri dish inside which the channel system was fabricated and later perfused directly. Figure 3 shows the different complexity levels of the fabricated devices.

### 3.3. Perfusion

**Figure 4:**
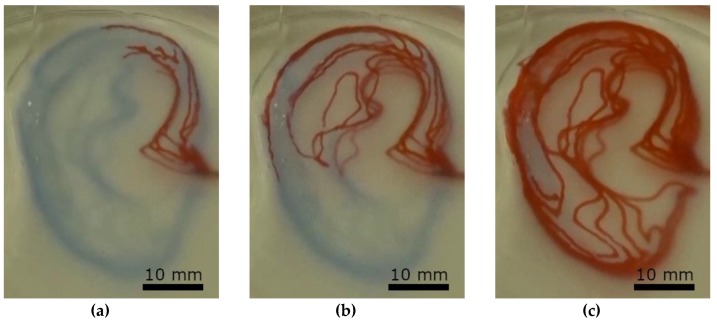
Perfusion test of the earlobe-shaped channel (Figure 3c) system at three different stages by manual injection of red ink (Pelikan 4001). (**a**) after initial injection, (**b**) half full channel system, (**c**) full channel system.

Perfusion of the microfluidic devices was tested using ink, which was injected manually with a syringe. The results are shown in Figure 4, as well as in the video in supplementary material.

### 3.4. Morphology and Structure

**Figure 5:**
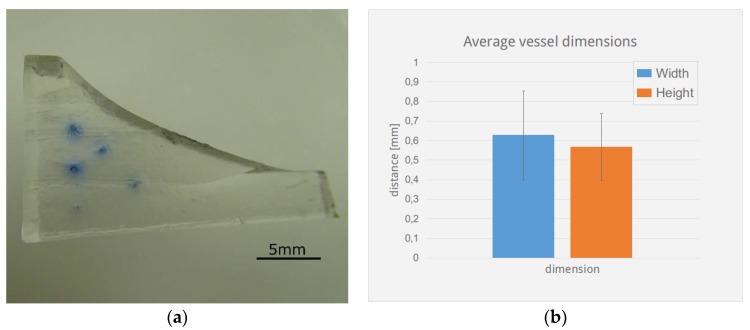
Meniscus channel cross-section. 5 meniscus structures (Figure 3b) were cut perpendicular to the channels and the vessel diameter in the lateral (blue) and vertical directions (orange) were measured under a stereomicroscope with the aid of ImageJ. (**a**) shows a sample section with channel cross-sections and stains from the diffused ink. (**b**) shows a bar-chart with average values and standard deviation.

To obtain a homogeneous gelatine particle size and more precise control over printing resolution, granulated gelatine was finely ground and sifted. Decreasing grain size increases printing resolution of the vessels, thus channels with an average diameter of 0.6 mm in a slurry made from 90–200 micron sized particles showed a standard deviation in the x and y directions of 0.2 mm, see Figure 5. Decreasing grain size however also reduces the stability of the final scaffold, thus meniscus structures made from particles with <90 um size did not exhibit self-supporting stability when made with the described method.

To increase the stability of the fabricated structures additional cross-linking would be an option. Studies have shown that gelatine scaffolds cross-linked with Glutaraldehyde and treated with sodium borohydride exhibit significantly increased stability, even in cell culture conditions (37 °C, 5% CO_2_) and provide sufficient biocompatibility [11,12].

### 3.5. Alginate Based Structures

**Figure 6:**
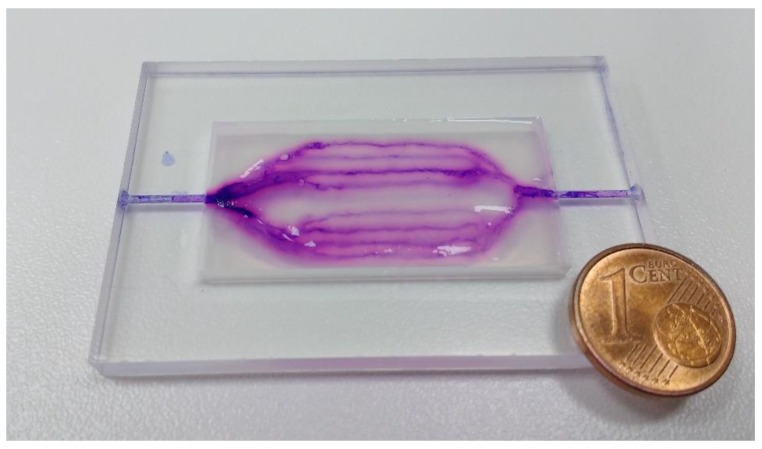
Alginate-based microfluidic device in the shape of 2D branched structures. Perfusion was tested using violet ink (Pelikan 4001), residues of which are visible in the photograph. The same sample frame and fabrication parameters were used here, as shown in Figure 3a.

In addition, fabrication of hydrogel microfluidic devices can be performed using other biocompatible and biopolymers which can be translated into granulated form. This was shown by fabricating perfusable hydrogel structures using alginate tested in a simple flat geometry, results are shown in Figure 6.

This demonstrates that this method is also useful with other polymers, which allow the fabrication of beads, increasing the range of applications. The FRESH method was already shown to produce vascular structures where vessel walls were fabricated, which; however, sets a limit on the resolution and fabrication complexity and finer vessels (≤1 mm) are difficult to manufacture in this manner. The proposed method allows fabricating vessels with a diameter comparable to the nozzle. Using gelatine granules and G27 nozzles (0.21 mm inner diameter, 0.41 mm outer diameter), 0.6 mm thick vessels could be produced, which should be optimized by improving matrix composition. By increasing the range of materials which can be used with this technique, diffusion and other properties will be possible to regulate. The described method allows the fabrication of microfluidic devices from any hydrogel which allows fabrication of beads/granulate in the appropriate range of particle-size ≤300 µm. Internal channel pressure was not measured in this work; however, we did observe a certain variation in flow velocity between channels in the ear-lobe shaped device. This can be explained by varying channel pressures which according to Hagen–Poiseuille’s law arise due to differences in channel length. To confirm this and produce a more detailed analysis of flow behaviour inside channels which were fabricated in the described manner, we suggest the use of a controlled pumping system, coupled with visual analysis for obtaining quantitative data.

## 4. Conclusions

In summary, we describe a method for the fabrication of perfusable freeform microfluidics, created from hydrogels based on biopolymers. By grinding and sifting particle size was controlled, and together with a bonding solution composed of 100 mM CaCl_2_ and 10% gelatine. Using this method it was possible to create 3D structures, stable enough to support themselves as well as complex channel geometries which were evenly perfusable. While additional work is required to engineer de-novo vascular tissues, with this method it will be much simpler to achieve. Thus, it holds enormous potential for the fabrication of vascularized structures as the freeform properties allow creating perfusable channels in not only various controlled paths, but also in differently shaped matrices. The described method creates a new approach towards the fabrication of vascular grafts; however, much work is still required in this direction. Vessel resolution as well as shape fidelity need to be improved, the spectrum of usable materials also needs to be expanded, adjusting biochemical, as well as structural properties of the devices.

## Supplementary Materials

The following are available online at http://www.mdpi.com/1996-1944/11/12/2529/s1, Video S1: A video of perfusion, as shown in Figure 4.
